# The role of radiotherapy in metaplastic breast cancer: a propensity score-matched analysis of the SEER database

**DOI:** 10.1186/s12967-019-2069-y

**Published:** 2019-09-23

**Authors:** Yongfeng Li, Meng Chen, Barbara Pardini, Mihnea P. Dragomir, Anthony Lucci, George A. Calin

**Affiliations:** 1 Department of Breast Surgery, Institute of Cancer and Basic Medicine (ICBM), Chinese Academy of Science; Cancer Hospital of the University of Chinese Academy of Sciences; Zhejiang Cancer Hospital, Hangzhou, 310022 Zhejiang People’s Republic of China; 2 Department of Experimental Therapeutics, The University of Texas MD Anderson Cancer Center, South Campus Research Building 4, 1901 East Road, Houston, TX 77054 USA; 3 Italian Institute for Genomic Medicine, Turin, Italy; 4 Department of Surgery, Fundeni Clinical Hospital, 22328 Bucharest, Romania; 5 The Research Center for Functional Genomics, Biomedicine and Translational Medicine, Iuliu Hatieganu University of Medicine and Pharmacy, 400012 Cluj Napoca, Romania; 6 Department of Breast Surgical Oncology, The University of Texas MD Anderson Cancer Center, 1400 Pressler St, Unit 1484, Houston, TX 77030 USA; 7 Center for RNA Interference and Non-coding RNAs, The University of Texas MD Anderson Cancer Center, Houston, TX 77054 USA

**Keywords:** Breast neoplasms, Metaplastic breast cancer, Radiotherapy, Prognosis, Survival

## Abstract

**Background:**

Only few studies, with small patient cohorts, have evaluated the effect of radiotherapy (RT) for metaplastic breast cancer (MBC). Hence, it is important to investigate the role of RT in MBC survival using a large population-database.

**Methods:**

A retrospective cohort study using the Surveillance, Epidemiology, and End Results (SEER) from 1973 to 2015 was performed. We compared MBC patients with or without RT for overall survival (OS) and breast cancer-specific survival (BCSS) using univariate and multivariate Cox proportional hazard regressions before and after propensity score matching (PSM).

**Results:**

From a total of 2267 patients diagnosed with MBC between 1998 and 2015, 1086 (47.9%) received RT. In the multivariate analysis before PSM, RT provided a better OS (HR 0.73; 95% CI 0.61–0.88; p = 0.001) and BCSS (HR 0.71; 95% CI 0.58–0.88; p = 0.002). Multivariate analyses after PSM (n = 1066) confirmed that patients receiving RT (n = 506) survived longer than those without RT (OS, HR 0.64; 95% CI 0.51–0.80; p < 0.001 and BCSS, HR 0.64; 95% CI 0.50–0.83; p = 0.001). A longer OS was observed when RT was given to older patients (p = 0.001) and in case of large tumor size (p = 0.002). Intriguingly, patients with N0 stage showed better OS after RT (HR 0.69, P = 0.012).

**Conclusions:**

Our findings support the beneficial effect of RT for MBC patients. In particular, older patients or with large tumor size have a greater survival benefit from RT. In conclusion, we have assessed the importance of the use of RT in MBC as survival factor and this could lead to the development of guidelines for this rare sub-type of tumors.

## Background

Metaplastic breast cancer (MBC) is a rare pathologic entity of the mammary gland accounting for about 0.2–2% of breast cancer diagnoses and is generally associated with poor overall survival (OS) [1–3]. In 2010, MBC was defined as a unique histologic subtype by the World Health Organization [4]. The histologic classification of MBC is primarily based on the morphology of tumor cell types: purely epithelial or mesenchymal components, or a mixture of both [5]. Because of the increased cognizance of MBC by pathologists, lately there has been a rise in diagnoses [6, 7]. In the past decade, four independent databases confirmed the worse prognosis of MBC compared with non-MBC [7–10]. However, in light of its rarity, there are no association-endorsed treatment guidelines specific to the management of MBC. In the recently revised National Comprehensive Cancer Network (NCCN) guidelines, metaplastic carcinoma, defined as more than 10% of the tumor phenotype, is an independent prognostic variable; however, the guidelines used for its treatment are the same as for infiltrating ductal carcinoma (IDC) [11]. Compared with IDC, MBC tumors are often in a more advanced T stage, less likely to have nodal involvement, more likely to be hormone receptor negative, and of higher grade. A recent case–control study demonstrated that the proportion of triple negative breast cancer (TNBC) in patients with stage I-III MBC is significantly higher than in IDC patients with the same stage (64.1% vs 12%, p < 0.001) [12]. Even when restricted to patients with TNBC, survival disparities persist between MBC and IDC [8].

Because of the high incidence of hormone receptor negativity in MBC, the majority of these patients receive systemic therapy after surgical treatment [13]. Endocrine therapy is unlikely to influence survival. On the other hand, Cimino et al. [14] showed that adjuvant chemotherapy was associated with improved OS of patients with MBC, although the effect was limited to early-stage cases. Multiple other reports have demonstrated that MBC have a poorer response to chemotherapy regimens when compared to IDC [15–20]. Regarding the effectiveness of radiotherapy (RT) for MBC, there is a limited number of studies and the investigated patient cohorts are generally small. Tseng and Martinez [10] studied a cohort of MBC patients treated between 1988 and 2006 and concluded that the use of adjuvant RT independently associates with improved OS. Similar results were reported in another study, indicating an improvement in local–regional recurrence (LRR) (p = 0.009) and OS (p < 0.001) after RT [21].

Thus, adjuvant RT should be explored as an approach to improve the dismal outcome of MBC. Indeed, precise guidelines are needed regarding the administration of adjuvant RT.

For these reasons, we analyzed a large database from the Surveillance, Epidemiology, and End Results (SEER) registry, through conventional methods and a propensity score matching (PSM) approach to investigate the impact of postoperative RT and clinicopathologic factors of MBC on patient prognostics.

## Methods

### Study population and data sources

The database from the National Cancer Institute’s SEER program was queried to build our study cohort. The SEER database includes 18 registries covering approximately 28% of the U.S. population and contains basic demographics and some clinical characteristics [22]. We used the SEER database, to include all participants diagnosed with microscopically confirmed MBC between the years 1973–2015. Metaplastic histology was identified with SEER ICD-0-3 codes: 8052, 8070–8072, 8074, 8560, 8571, 8572, 8575, and 8980. All patients diagnosed on autopsy or death certificate, or that presented stage IV MBC, with multiple primary lesions, or that received neoadjuvant RT were excluded from the study. The following clinicopathological factors were extracted from the SEER database: age at diagnosis; marital status; race; TNM stage; tumor grade; hormone receptor status; T stage; N stage and treatment data including surgery for the primary site, chemotherapy record, and adjuvant radiotherapy.

### Survival analysis and propensity matching

PSM is a tool for decreasing selection bias in non-randomized studies and achieving balance covariates across treatment groups. The propensity score is the conditional probability of assignment to a particular treatment given a vector of observed covariates [23]. PSM permits the exclusion of bias factors that predict a type of treatment rather than the treatment per se. We created a matched dataset using PSM, using age (over and equal or under 60 years old), marital status, race, T stage, N stage, tumor grade, estrogen receptor (ER) status, progesterone receptor (PR) status, and treatment options including surgery (lumpectomy or mastectomy) and chemotherapy (yes versus no) as covariates. Then, PSM was performed using 1:1 nearest neighbor matching to create a matched pair between the RT group and the No RT group. A Chi square test for categorical variables was used to compare across groups.

### Statistical analysis

We employed univariate and multivariate Cox proportional hazard models to identify factors associated with improved OS and breast cancer-specific survival (BCSS), using results reported as hazard ratio (HR) and 95% confidence intervals (CI). OS was defined as the time from diagnosis to death or last follow-up. Patients with BCSS were identified using the cause of death provided by the death certificate. In order to account for missing values, multiple imputation methods using polytomous logistic regression were applied by MICE package in R software [24] and pooled the modeled data for a complete data set. The Kaplan–Meier method was used to estimate the survival curve and log-rank test was performed for comparison of survival between the nominal variables. All statistical analyses were conducted using R software (ver.3.5.1) and SPSS statistical software (ver.24.0) with a two-sided *p* value < 0.05 considered statistically significant.

## Result

### Patient characteristics

**Fig. 1:**
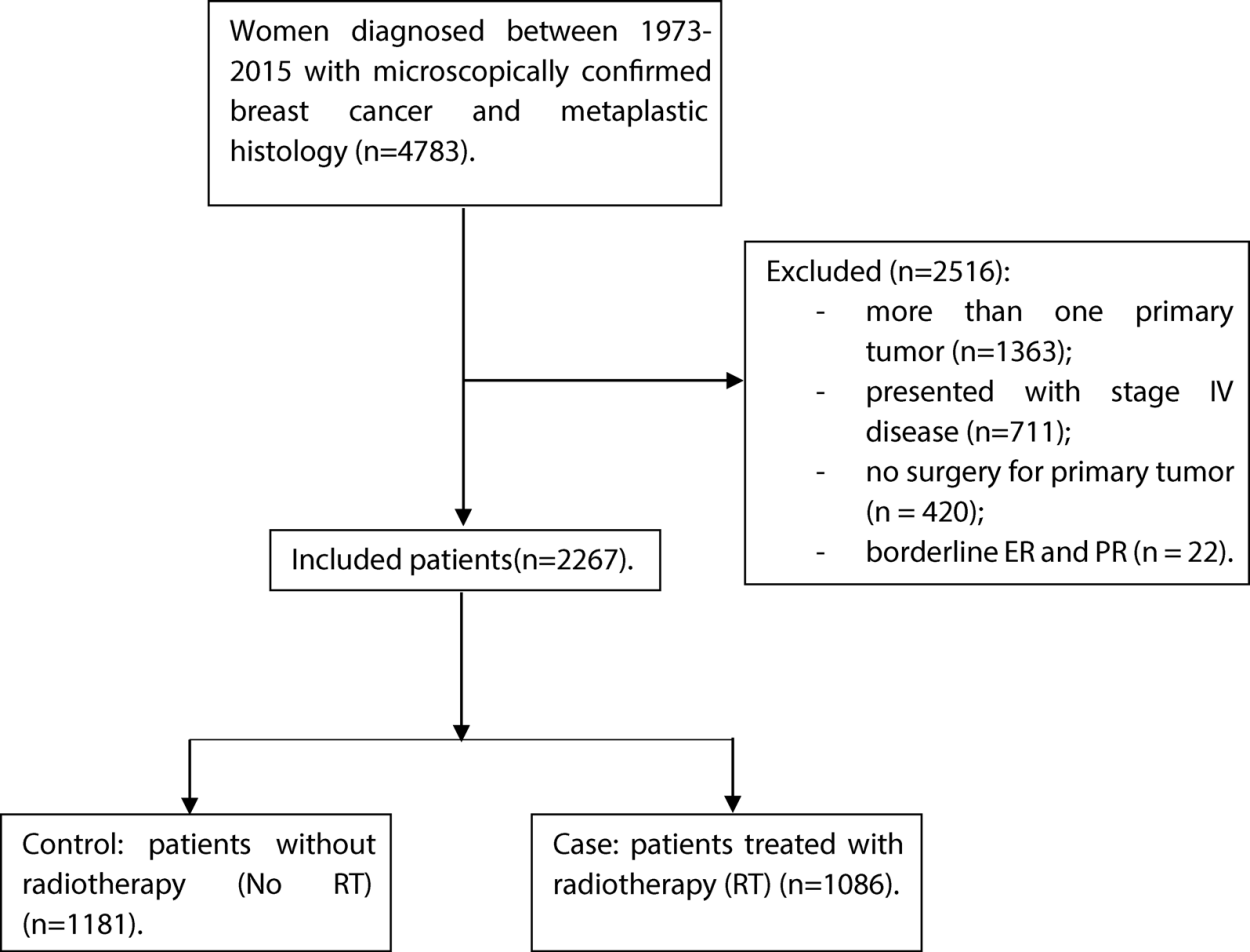
Flow diagram of study cohort selection

Overall, 2267 patients who received treatment for MBC were identified from the SEER database. We divided the patients into two distinct groups, those who received RT (case, n = 1086) and those who did not receive adjuvant RT (No RT-control, n = 1181) (Fig. 1). Because multiple clinical parameters were necessary for this study, only patients diagnosed with MBC during 1998–2015 met the inclusion criteria (Additional file 1: Table S1). Clinical and pathologic characteristics of all MBC patients are presented in Additional file 2: Table S2. The median follow-up for all MBC patients was 44 months. MBC tumors were more commonly of high grade (G3/G4: 72.4%), although 10.9% had unknown tumor grade, and no lymph node involvement (76.8%), Patients were more commonly treated with mastectomy (58.3%). Breast-conserving surgery was carried out in 41.7% of patients. ER and PR were not expressed in 77.1% and 81.5% of MBC patients, respectively. Around 51.2% of patients underwent sentinel lymph node biopsy (SLNB). Given the significant differences between case and control groups, a PSM was used to balance the distribution of most demographic and clinical characteristics. After matching, no variables were significantly different between cases and controls (Table 1). Participants were predominately matched within the common region (Additional file 3: Figure S1A).

**Table 1:** Selected baseline characteristics for the SEER database study population divided per study groups before and after PSM

Characteristics	Before PSM (2267)			After PSM (1066)		
	No RT	RT	*P*-value	No RT	RT	*P*-value
Age at diagnosis			< 0.05			0.057
< 60	504 (42.7%)	592 (54.5%)		264 (49.5%)	296 (55.5%)	
≥ 60	677 (57.3%)	494 (45.5%)		269 (50.5%)	237 (44.5%)	
Ethnicity			*< 0.05*			0.667
White	929 (78.7%)	825 (76.0%)		402 (75.4%)	408 (76.5%)	
Black	163 (13.8%)	190 (17.5%)		95 (17.8%)	85 (15.9%)	
Others	89 (7.5%)	71 (6.5%)		36 (6.8%)	40 (7.5%)	
Marital status			0.263			0.736
Single	185 (15.7%)	151 (13.9%)		86 (16.1%)	81 (15.2%)	
Married	996 (84.3%)	935 (86.1%)		447 (83.9%)	452 (84.8%)	
Grade			0.539			0.472
G1	64 (5.4%)	56 (5.2%)		23 (4.3%)	24 (4.5%)	
G2	173 (14.6%)	137 (12.6%)		66 (12.4%)	69 (12.9%)	
G3	888 (75.2%)	839 (77.3%)		416 (78.0%)	400 (75.0%)	
G4	56 (4.7%)	54 (5.0%)		28 (5.3%)	40 (7.5%)	
ER status			0.413			0.456
Negative	979 (82.9%)	885 (81.5%)		451 (84.6%)	441 (82.7%)	
Positive	202 (17.1%)	201 (18.5%)		82 (15.4%)	92 (17.3%)	
PR status			0.058			0.192
Negative	1044 (88.4%)	930 (85.6%)		475 (89.1%)	460 (86.3%)	
Positive	137 (11.6%)	156 (14.4%)		58 (10.9%)	73 (13.7%)	
Stage T			*< 0.05*			0.457
≤ 5 cm	937 (79.3%)	814 (75.0%)		373 (70.0%)	385 (72.2%)	
> 5 cm	244 (20.7%)	272 (25.0%)		160 (30.0%)	148 (27.8%)	
Stage N			0.090			0.202
N0	929 (78.7%)	811 (74.7%)		381 (71.5%)	382 (71.7%)	
N1	173 (14.6%)	195 (18.0%)		96 (18.0%)	112 (21.0%)	
N2	51 (4.3%)	45 (4.1%)		35 (6.6%)	22 (4.1%)	
N3	28 (2.4%)	35 (3.2%)		21 (3.9%)	17 (3.2%)	
Stage TNM			*< 0.05*			0.848
I	247 (20.9%)	283 (26.1%)		109 (20.5%)	115 (21.6%)	
II	779 (66.0%)	605 (55.7%)		314 (58.9%)	314 (58.9%)	
III	155 (13.1%)	198 (18.2%)		110 (20.6%)	104 (19.5%)	
Breast operation			*< 0.05*			0.667
Lumpectomy	254 (21.5%)	692 (63.7%)		248 (46.5%)	240 (45.0%)	
Mastectomy	927 (78.5%)	394 (36.3%)		285 (53.5%)	293 (55.0%)	
Chemotherapy			*< 0.05*			0.111
Not done/unknown	548 (46.4%)	254 (23.4%)		176 (33.0%)	151 (28.3%)	
Done	633 (53.6%)	832 (76.6%)		357 (67.0%)	382 (71.7%)	
Axilla LN operation			*0.050*			0.425
SLNB	581 (49.2%)	580 (53.4%)		261 (49.0%)	247 (46.3%)	
ALND	600 (50.8%)	506 (46.6%)		272 (51.0%)	286 (53.7%)	

*SLNB* Sentinel lymph node biopsy, *ALND* axillary lymph node dissection

Significant results are in italic

### Survival analyses in the whole SEER cohort

**Fig. 2:**
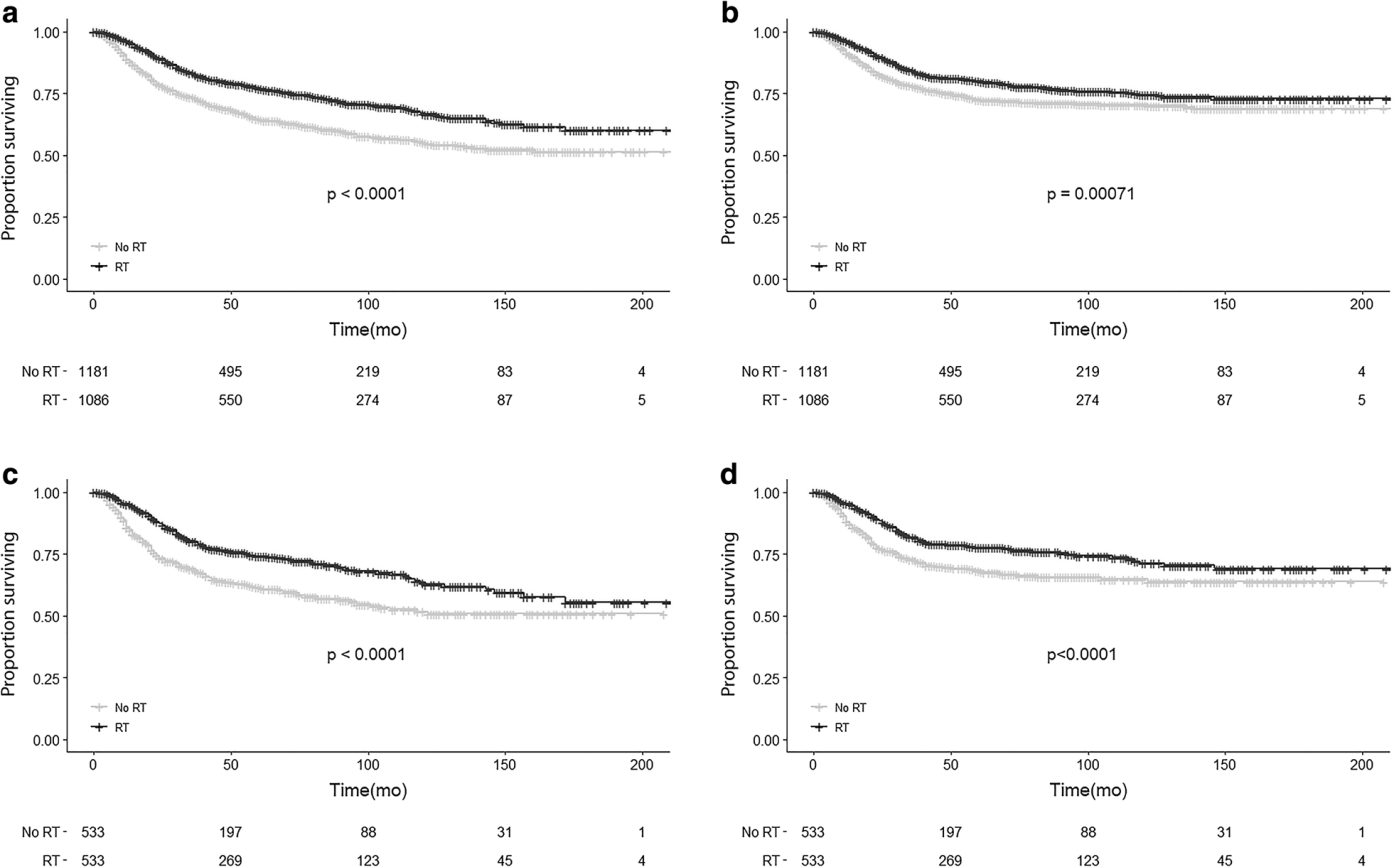
OS and BCSS of MBC patients displayed as Kaplan–Meier curve stratified according to RT. **a** OS curves of RT cohort versus no RT cohort before PSM. **b** BCSS curves of RT cohort versus No RT cohort before PSM. **c** OS curves of RT cohort versus no RT cohort after PSM. **d** BCSS curves of RT cohort versus No RT cohort after PSM

OS of the entire cohort was 70.7% at 5 years and 61.0% at 10 years while the BCSS of was 76.3% at 5 years and 72.4% at 10 years. All the baseline characteristics and selected variables were included in univariate and multivariate analyses in relation to both OS and BCSS (Table 2). Patients who underwent mastectomy were found to receive less RT than patients undergoing breast conserving therapy (Additional file 3: Figure S1B), despite a much higher rate of tumors > 5 cm (Additional file 3: Figure S1C). As expected, increased age, higher N stage, and larger tumor size were associated with worse OS and BCSS, while receiving RT was strongly associated with better survival (OS: HR 0.73; 95% CI 0.61–0.88; p = 0.001 BCSS: HR 0.71; 95% CI 0.58–0.88; p = 0.002). In fact, in patients receiving RT, OS was 77.1% at 5 years and 66.9% at 10 years versus respectively 64.6% and 55.3% in patients not receiving RT (Fig. 2a). BCSS for patients receiving RT was 80.1% at 5 years and 74.5% at 10 years compared with 72.6% and 70.5% in patients not receiving RT (Fig. 2b). Chemotherapy was only associated with improved OS, but not BCSS in multivariate analysis (Table 2).

**Table 2:** Univariate and multivariate analyses of OS and BCSS for the MBC variables included in the study before PSM

Characteristics	Univariate				Multivariate			
	OS		BCSS		OS		BCSS	
	HR (95% CI)	*P*-value	HR (95% CI)	*P*-value	HR (95% CI)	*P*-value	HR (95% CI)	*P*-value
Age at diagnosis (< 60 as ref.)								
≥ 60	1.92 (1.63–2.26)	*< 0.01*	1.23 (1.02–1.48)	*<* *0.05*	1.81 (1.51–2.16)	*< 0.01*	1.36 (1.11–1.66)	*< 0.001*
Race (white as ref.)								
Black	1.02 (0.82–1.27)	0.843	1.12 (0.88–1.43)	0.364	1.01 (0.81–1.26)	0.958	0.97 (0.76–1.25)	0.828
Others	0.91 (0.65–1.26)	0.583	0.98 (0.68–1.42)	0.922	0.95 (0.69–1.32)	0.763	0.97 (0.67–1.41)	0.872
Marital status (single as ref.)								
Married	0.92 (0.74–1.14)	0.449	0.78 (0.62–1.00)	*< 0.05*	0.93 (0.74–1.17)	0.54	0.92 (0.71–1.18)	0.506
Grade (G1 as ref.)								
G2	1.23 (0.79–1.93)	0.36	1.08 (0.61–1.92)	0.786	1.08 (0.69–1.69)	0.747	0.93 (0.52–1.65)	0.801
G3	1.5 (1.01–2.25)	< *0.05*	1.77 (1.07–2.92)	< *0.05*	1.18 (0.78–1.77)	0.438	1.14 (0.69–1.9)	0.605
G4	1.94 (1.20–3.15)	< *0.01*	2.50 (1.40–4.49)	< *0.01*	1.56 (0.96–2.54)	0.076	1.65 (0.92–2.98)	0.095
ER status (negative as ref.)								
Positive	0.9 (0.73–1.12)	0.36	0.88 (0.68–1.13)	0.318	0.88 (0.68–1.14)	0.316	0.8 (0.59–1.08)	0.146
PR status (negative as ref.)								
Positive	0.8 (0.62–1.03)	0.077	0.87 (0.66–1.16)	0.339	0.81 (0.60–1.09)	0.162	0.95 (0.68–1.34)	0.779
Stage T (≤ 5 cm as ref.)								
> 5 cm	3.65 (3.11–4.28)	< *0.01*	4.55 (3.79–5.46)	< *0.001*	2.92 (2.44–3.49)	< *0.01*	3.44 (2.79–4.24)	< *0.001*
Stage N (N0 as ref.)								
N1	1.79 (1.48–2.17)	< *0.01*	2.35 (1.89–2.91)	< *0.001*	1.55 (1.26–1.91)	< *0.01*	1.78 (1.41–2.25)	< *0.001*
N2	3.45 (2.60–4.59)	< *0.01*	4.01 (2.91–5.52)	< *0.001*	2.55 (1.88–3.46)	< *0.01*	2.49 (1.76–3.51)	< *0.001*
N3	3.86 (2.78–5.36)	< *0.01*	5.03 (3.54–7.17)	< *0.001*	2.91 (2.06–4.11)	< *0.01*	3.33 (2.29–4.84)	< *0.001*
Breast operation (lumpectomy as ref.)								
Mastectomy	2.56 (2.14–3.06)	< *0.01*	2.75 (2.22–3.41)	< *0.001*	1.3 (1.05–1.61)	< *0.05*	1.32 (1.02–1.70)	< *0.05*
Chemotherapy (not done/unknown as ref.)								
Done	0.62 (0.53–0.72)	< *0.01*	0.99 (0.82–1.2)	*0.934*	0.71 (0.60–0.85)	< 0.001	1.02 (0.82–1.27)	0.848
Radiotherapy (not done as ref.)								
Done	0.62 (0.53–0.73)	< *0.001*	0.73 (0.61–0.88)	< *0.001*	0.73 (0.61–0.88)	*0.001*	0.71 (0.58–0.88)	< *0.01*
Axilla LN operation (SLNB as ref.)								
ALND	1.91 (1.62–2.25)	< *0.001*	2.18 (1.79–2.65)	< *0.001*	1.17 (0.97–1.42)	0.096	1.14 (0.91–1.43)	0.254

*SLNB* Sentinel lymph node biopsy, *ALND* axillary lymph node dissection

Significant results are in italic

In order to assess the influence of RT regimen and chemotherapy regimen on our results, we determined the time points when significant changes in BC therapy took place and we split our initial cohort into two cohorts, before and after the changes in therapy. The most significant changes in the RT regimen for breast cancer patients occurred during the period 1997–1999 [25–27]. In the present study, the majority (96.33%) of the MBC patients were diagnosed after 2000 (Additional file 1: Table S1), implying that the radiotherapy received by these patients was mostly homogenous.

On the other hand, around 2005 we assisted to a significant change in breast cancer treatment which consisted in the addition of taxanes (docetaxel and paclitaxel) to the adjuvant chemotherapy regimens [28, 29]. Therefore, we decided to divide the cohort in two time periods and repeat the analysis to test the effect of RT for MBC in the 2 different periods (Group 1: from 1998 to 2005 and Group 2: from 2006 to 2015). Interestingly, for both groups we obtained a better OS and BCSS if radiotherapy was performed (similar to the results on the whole cohort) (Additional file 4: Figure S2 A–D). Despite the changes in the chemotherapy regimen, the addition of radiotherapy to MBC is still beneficial. There was in fact a better OS for the 2006–2015 cohort (P < 0.0001; Additional file 4: Figure S2C) than the 1998–2005 cohort (P = 0.013, Additional file 4: Figure S2A) and the BCSS was minimally improved in the 2006–2015 cohort (1998–2005 Cohort: P = 0.0024; 2006–2015 Cohort: P = 0.0019; Additional file 2: Table S2 B and D). This could be due in part because of the taxanes, which are known to increase the radio-sensitivity of cancer cells in vitro [30].

### Survival analysis in propensity score-matched cohort

In the matched cohort, univariate analysis revealed similar prognostic factors for OS and BCSS to the results of unmatched cohort: age, PR status, marital status, larger tumor size, higher N stage and, axillary lymph node dissection (ALND). Only age did not result a prognostic factor for BCSS. Survival curves according to the RT are shown in Fig. 2c, d. Multivariate analysis also showed that patients receiving RT survived significantly longer than those without RT (5-year OS, 74.2% vs 61.5% p < 0.001; BCSS, 71.4% vs 64.9% p < 0.001). Additionally, age, marital status, larger tumor size, and higher N stage were maintained as prognostic factors for OS and BCSS. Results of survival analysis in the propensity-matched cohort are summarized in Table 3.

**Table 3:** Univariate and multivariate analyses of OS and BCSS for the MBC variables included in the study after PSM

Characteristics	Univariate				Multivariate			
	OS		BCSS		OS		BCSS	
	HR (95% CI)	*P*-value	HR (95% CI)	*P*-value	HR (95% CI)	*P*-value	HR (95% CI)	*P*-value
Age at diagnosis (< 60 as ref.)								
≥ 60	1.49 (1.20–1.85)	< *0.001*	1 (0.78–1.289)	0.987	1.6 (1.26–2.05)	< *0.001*	1.23 (0.94–1.62)	0.138
Race (white as ref.)								
Black	1.18 (0.89–1.60)	0.243	1.22 (0.89–1.68)	0.22	1.03 (0.77–1.39)	0.842	0.95 (0.68–1.32)	0.755
Others	0.96 (0.61–1.49)	0.842	1.07 (0.66–1.73)	0.798	1.09 (0.69–1.71)	0.723	1.06 (0.65–1.74)	0.813
Marital status (single as ref.)								
Married	0.7 (0.53–0.94)	< *0.05*	0.62 (0.46–0.84)	< *0.01*	0.68 (0.50–0.91)	*0.01*	0.68 (0.49–0.95)	< *0.05*
Grade (G1 as ref.)								
G2	0.98 (0.48–2.01)	0.958	0.8 (0.33–1.93)	0.617	0.96 (0.47–1.98)	0.918	0.8 (0.33–1.94)	0.62
G3	1.58 (0.84–2.97)	0.155	1.8 (0.85–3.82)	0.127	1.23 (0.65–2.32)	0.529	1.26 (0.59–2.68)	0.557
G4	1.34 (0.64–2.80)	0.439	1.58 (0.67–3.77)	0.299	1.34 (0.64–2.81)	0.443	1.44 (0.60–3.44)	0.411
ER status (negative as ref.)								
Positive	0.82 (0.60–1.14)	0.239	0.84 (0.59–1.21)	0.353	0.83 (0.57–1.21)	0.331	0.76 (0.50–1.15)	0.19
PR status (negative as ref.)								
Positive	0.63 (0.43–0.93)	< *0.05*	0.77 (0.51–1.15)	0.204	0.67 (0.43–1.04)	0.074	0.89 (0.56–1.42)	0.627
Stage T (≤ 5 cm as ref.)								
> 5 cm	3.84 (3.08–4.78)	< *0.001*	4.41 (3.44–5.66)	< *0.001*	2.99 (2.31–3.86)	< *0.001*	3.18 (2.39–4.24)	< *0.001*
Stage N (N0 as ref.)								
N1	1.56 (1.20–2.05)	*0.001*	2.02 (1.51–2.71)	< *0.001*	1.21 (0.91–1.61)	0.191	1.43 (1.04–1.95)	< *0.05*
N2	2.91 (2.02–4.20)	< *0.001*	3.29 (2.19–4.95)	< *0.001*	1.84 (1.24–2.72)	< *0.01*	1.82 (1.17–2.82)	< *0.01*
N3	3.27 (2.14–5.00)	< *0.001*	4.04 (2.55–6.40)	< *0.001*	2.2 (1.39–3.48)	*0.001*	2.41 (1.46–3.98)	*0.001*
Breast operation (lumpectomy as ref.)								
Mastectomy	2.6 (2.04–3.31)	< *0.001*	3.18 (2.38–4.25)	< *0.001*	1.46 (1.08–1.98)	< *0.05*	1.52 (1.06–2.18)	< *0.05*
Chemotherapy (not done/unknown as ref.)								
Done	0.84 (0.67–1.05)	0.12	1.35 (1.02–1.80)	< *0.05*	0.75 (0.58–1.00)	< *0.05*	1.08 (0.79–1.48)	0.617
Radiotherapy (not done as ref.)								
Done	0.62 (0.50–0.77)	< *0.001*	0.65 (0.51–0.84)	< *0.001*	0.64 (0.51–0.80)	< *0.001*	0.64 (0.50–0.83)	*0.001*
Axilla LN operation (SLNB as ref.)								
ALND	1.89 (1.49–2.39)	< *0.001*	2.22 (1.68–2.92)	< *0.001*	1.17 (0.89–1.54)	0.254	1.16 (0.85–1.60)	0.347

*SLNB* sentinel lymph node biopsy, *ALND* axillary lymph node dissection

Significant results are in italic

### Exploratory subgroup analysis assessing the benefit of RT according to the clinical characteristics

**Fig. 3:**
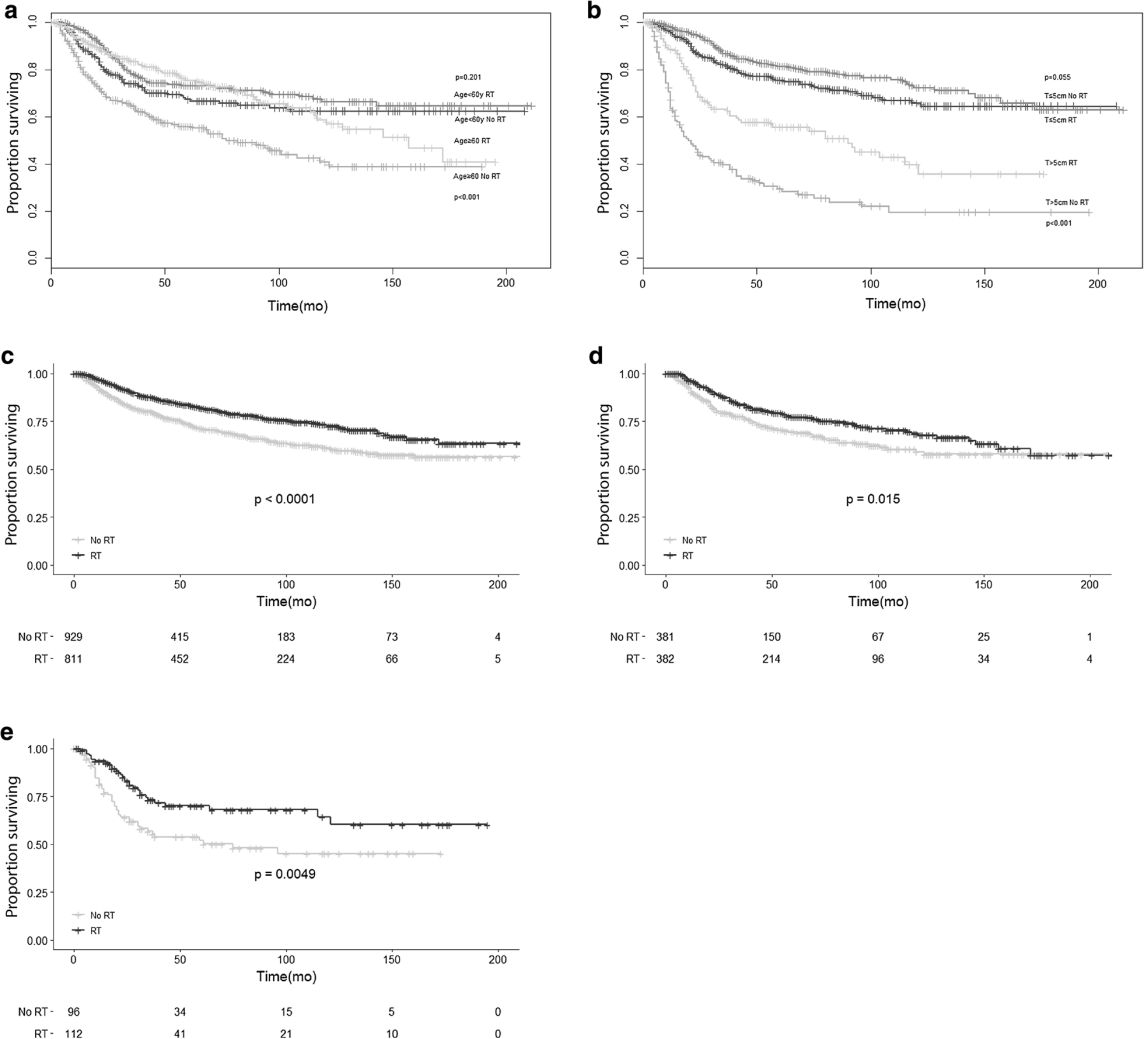
OS of MBC patients displayed as Kaplan–Meier curves according to RT for different patient subgroups: **a** age ≥ 60 years and age < 60 years subgroup with or without RT after PSM. **b** Tumor size ≤ 5 cm and a > 5 cm subgroup with or without RT after PSM. **c** N0 subgroup with or without RT before PSM. **d** N0 subgroup with or without RT after PSM. **e** N1 subgroup with or without RT after PSM

To detect precise subgroups of patients that could benefit from RT, PSM was performed for each subgroup including age, tumor size, N stage, breast operation, and ALND as covariates. A significantly increased OS was observed when RT was given to older patients (≥ 60 years old) (HR 0.614, P = 0.001) and those with larger tumor size (HR 0.593, P = 0.01) (Fig. 3a, b). In the subgroup of N0 stage, RT was also associated with an improvement of OS (Fig. 3c). After PSM, RT maintained the significant survival advantage in the N0 stage subgroup (Fig. 3d). MBC patients who received RT also had better survival in N1 stage subgroup (Fig. 3e). RT could reduce the risk of death by 58.0% for patients with N1 stage, while for patients with N0 stage the reduction was only 30.6%. Patients receiving breast-conserving surgery and RT demonstrated 43.7% decrease in death from any cause when compared with patients receiving mastectomy and RT, these last showing a reduction of only 25.7%. Patients who underwent SLNB and ALND had similar benefit from RT (42.9% vs 31.9.0% reduction of deaths, respectively) (Additional file 5: Figure S3).

## Discussion

To our knowledge, this is the first population-based study using PSM analysis to assess the role of RT in treating MBC. In this study, significant improvements in survival were observed for patients treated with RT, especially when elderly (≥ 60 years), and with large tumor size. The beneficial effect on survival observed in the SEER database highlights the importance of RT in the management of MBC.

As a rare breast cancer subtype, the optimal treatment options for MBC are relatively unknown. The role of RT in improving survival for locally advanced IDC and post breast-conserving operations has been established well [8, 31]. However, for MBC the effectiveness of RT is yet to be defined. Although MBC is more aggressive than IDC, overall the use of RT is lower in the MBC population compared to IDC (48.3% MBC vs 54.3% IDC, P = 0.0001) [8]: in fact, only 62% of MBC patients who undergo lumpectomy received RT, while post-lumpectomy RT is a standard therapy for treating IDC patients [10]. In our study, 47.9% of MBC received RT, and the treatment was given to 73.2% of patients who received a breast-conserving operation. Rakha et al. [32] reported no association between RT and survival outcomes in patients with MBC, but several other studies demonstrated that the use of adjuvant RT independently associates with improved survival [8]. In a study on 1501 MBC patients, RT was found to be associated with better overall and disease-free survivals [10]. Another cohort study showed that RT was independently associated with better survival of MBC patients (HR 0.81, 95% CI 0.78–0.84). Similar results were reported in a case series study that indicated an improvement in the OS of MBC patients after RT [3]. In our study, univariate and multivariate results demonstrated that RT was independently associated with an improvement in OS and BCSS and the results were independent of the changes that occurred in chemotherapy regimen over the years. PSM analyses also confirmed these results.

Currently, RT practices on MBC are comparable with those on IDC [33]. Nevertheless, there are significant biological differences between MBC and IDC. Compared to IDC, MBC cells express lower levels of ER, PR, and HER-2 (a phenotype similar to TNBC) and express higher levels of Ki-67 and p53 [3, 6]. Molecular subtyping reveals that MBC tumors frequently display basal-like phenotypes. However, patients with triple-negative MBC have worse survival than patients with triple-negative IDC [15]. Moreover, patients with MBC have larger, higher-grade tumors with less involvement of the regional lymph nodes than IDC subjects [33, 34]. Additionally, MBC tends to disseminate hematogenously rather than lymphatic [13].

Wargot et al. [35] showed that MBC has an incidence of axillary lymph node metastasis ranging from 6 to 26%, depending on the subtype of MBC. Our study reported that only 23.2% of MBC had lymph node involvement. Since in MBC there is a high potential for metastatic spread to the lung and brain via blood vessels [36, 37], circulating tumor cells (CTC) may be playing a role in the metastatic progression. Some findings showed that the presence of CTC is an independent predictor of relapse and death in patients with operable breast cancer [38] and this could also be the case of MBC. In addition, several mutated genes were identified to correlate with the prognosis of patients with MBC. The principal immunohistochemical feature of MBC cells is the positive CD44 and the overexpression of the Yes-associated protein, both of which are stem cells markers [13, 39]. Genomic profiling has shown a down-regulation of the DNA repair pathways including *BRCA1*, *PTEN* and *TOP2A* [40, 41]. In some studies, up to 35% of MBC patients had *PIK3CA* mutations [42] and a PIK3CA inhibitor has also shown efficacy in improving outcomes of metastatic MBC [43]. A recent study showed that 46% of MBC expressed PD-L1 [44], opening up the possibility of trials using immune checkpoint inhibitors for MBC, as is now being tested in TNBC trials [45]. Finally, the presence of mesenchymal and sarcomatous elements also may explain the different biologic behavior and pattern of metastasis. Therefore, precise guidelines are needed regarding the administration of adjuvant RT.

Current guidelines recommend adjuvant RT for breast cancer patients with 4 or more metastatic axillary nodes, large primary tumor (> 5 cm) or after lumpectomy [46, 47]. MBC is characterized by large tumor size and rapid growth, hence RT should be considered. In the National Cancer Data Base (NCDB) study, a higher AJCC stage was common for MBC patients, despite having more lymph node-negative tumors. Hence, the tumor stage is strongly influenced by the tumor size. Compared with IDC patients, who at diagnosis have usually T1 tumors (65.2%), only 29.5% of MBC patients are in T1 stage at presentation [7]. A cohort study showed that RT is not useful for patients undergoing mastectomy with tumors < 5 cm or with < 4 metastatic axillary lymph nodes [10]. We confirmed these findings in our PSM analyses, after stratifying patients according to tumor size: RT was associated with improved OS in MBC patients with larger tumor size (> 5 cm). When stratified by N stage, we found that patients with both N0 and N1 could benefit from RT. Low likelihood of lymphatic involvement in MBC may be the cause of this result. However, RT could reduce the risk of death by 50.3% for patients with N2 stage, whereas for patients with N1 stage the reduction is only 26.2%. Due to the small sample sizes obtained after propensity matching, we did not find any survival benefit for stage N3 and N4.

Age over/equal 60 years at diagnosis was found to be a poor survival factor in multivariate analysis. Similar results have been reported in other population-based studies including SEER database (HR 2.9 95% CI 2.1–3.9) [9] and NCDB database (HR 1.018 95% CI 1.009–1.027) [48]. One of the reasons is that older women patients may not always get the most optimal treatment. Assuming older women may not handle treatment side effects as well as younger women, doctors tend to treat breast cancer in older women less aggressively. Therefore, the use of RT decreases with increasing age at diagnosis [49]. In our study, elderly (≥ 60 years old) received more rarely RT (54.5% vs 45.5%) compared to younger patients. Truong et al. [50] reported that radiation omission was significantly associated with increased relapse rates and poorer OS and BCSS. The highly aggressive MBC probably is one of the reasons for this negative outcome. Our subgroup analyses revealed that RT significantly improved OS in older MBC patients. Furthermore, the use of RT was shown to be associated with improved survival in patients, regardless of the surgical procedure performed. Interestingly, patients receiving breast-conserving surgery, and RT demonstrated a 43.7% decrease in death from any cause compared with patients receiving mastectomy and RT which showed a reduction of only 25.7%. Patients who underwent SLNB and ALND benefited similarly from RT (42.9% vs 31.9%).

Besides the large population included and the interesting results obtained for RT, we acknowledge some limitations to our study. One important limitation is the absence of information on systemic chemotherapy and endocrine therapy regimens due to the design of the SEER database. Missing these important parameters could lead to potential bias. Despite the large population, 2267 patients, selected for this study, the sample size was not big enough for more detailed subgroup analyses, such as age, ethnicity, and stage before PSM. The use of the PSM method might reduce the bias caused by the imbalanced distribution of the obtained covariates. In addition, the SEER database does not provide any data on some risk factors for breast cancer, such as smoking and menstrual status, which may contribute to additional study bias. Regarding RT in the SEER database, there is no information on the dose or intended target. Thus, we had no data about the radiation dose time, methods, intent, side effects, etc. which may all contribute to the survival. Interestingly, the large time interval of diagnosis for MBC patients may have been a bias considering that during this period, changes in chemotherapy regimen have been documented. For this reason, we divided the cohort in two time periods (1998–2005 and 2006–2015), before and after the addition of taxanes [28, 29]. We repeated the analysis to test the effect of RT for MBC in these 2 different sub-cohorts, giving a weight to the introduction of the use of taxanes in 2005. Interestingly, for both groups we obtained a better OS and BCSS if RT was performed, independently of the period of diagnosis [12]. Nevertheless, the SEER database usually has high completeness and is representative of the real patient population [22]. The results obtained are therefore robust from the statistical point of view and even after both multivariate and PSM analyses were performed, the OS and BCSS did not change appreciably.

## Conclusions

Based on our results, the MBC patients receiving RT resulted having a better BCSS and OS compared to MBC patients not treated with RT, in particular in presence of large tumors and elderly patients (≥ 60 years). Additionally, RT was associated with improved outcomes in patients with N0 stage, hence MBC patients with N0 stage could also benefit from RT. Further prospective studies with a sufficient sample size are needed to confirm these findings. In addition, although axillary node dissection is likely to add very little in terms of improved outcomes in MBC [48], omitting axillary node dissection could be evaluated in future studies to define the guidelines for MBC.

## Supplementary information

**Additional file 1: Table S1.** The year of diagnosis of patients from SEER database included in the study (n = 2267).

**Additional file 2: Table S2.** Clinicopathologic characteristics of all MBC patients (n = 2267).

**Additional file 3: Figure S1. A**. Plot of propensity score distribution before and after PSM; **B**. Breast operation by RT status before PSM; **C.** Breast operation by tumor size before PSM.

**Additional file 4: Figure S2.** OS and BCSS of MBC patients displayed as Kaplan–Meier curve stratified according to RT in two subgroups. **A.** OS curves of RT cohort versus no RT cohort after PSM diagnosed from 1998 to 2005. **B.** BCSS curves of RT cohort versus no RT cohort after PSM diagnosed from 1998 to 2005. **C.** OS curves of RT cohort versus no RT cohort after PSM diagnosed from 2006 to 2015. **D.** BCSS curves of RT cohort versus No RT cohort after PSM diagnosed from 2006 to 2015.

**Additional file 5: Figure S3.** Hazard ratio and 95% confidence interval for OS according to receiving RT for different subgroups of patients for several variables: age, tumor size, N stage, type of breast operation, axillary LN dissection.

## Data Availability

The datasets analyzed during this current study are available in SEER database to extract the eligible cases. The data are also available from the corresponding author on reasonable request.

## Abbreviations

RT: radiotherapy
MBC: metaplastic breast cancer
SEER: Surveillance, Epidemiology, and End Results
OS: overall survival
BCSS: breast cancer-specific survival
PSM: propensity score matching
IDC: infiltrating ductal carcinoma
TNBC: triple negative breast cancer
LRR: local-regional recurrence
ER: estrogen receptor
PR: progesterone receptor
HR: hazard ratio
CI: confidence intervals
SLNB: sentinel lymph node biopsy
ALND: axillary lymph node dissection
CTC: circulating tumor cells
NCDB: National Cancer Data Base
