# Supplementary material for: The role of radiotherapy in metaplastic breast cancer: a propensity score-matched analysis of the SEER database
Source: J Transl Med. 2019 Sep 23;17:318. doi: 10.1186/s12967-019-2069-y (PMC6757394; doi:10.1186/s12967-019-2069-y)
Supplement: Supplementary file 1 — Additional file 1: Table S1. The year of diagnosis of patients from SEER database included in the study (n = 2267). [file 12967_2019_2069_MOESM1_ESM.docx]

**Table S1.** The year of diagnosis of patients from SEER database included in the study (n=2267)

| Year of diagnosis | Patients treated with radiotherapy and included in the study |
| --- | --- |
| 1998 | 16 |
| 1999 | 28 |
| 2000 | 39 |
| 2001 | 94 |
| 2002 | 100 |
| 2003 | 117 |
| 2004 | 119 |
| 2005 | 131 |
| 2006 | 109 |
| 2007 | 138 |
| 2008 | 130 |
| 2009 | 138 |
| 2010 | 166 |
| 2011 | 184 |
| 2012 | 185 |
| 2013 | 181 |
| 2014 | 204 |
| 2015 | 188 |
