# Supplementary material for: The role of radiotherapy in metaplastic breast cancer: a propensity score-matched analysis of the SEER database
Source: J Transl Med. 2019 Sep 23;17:318. doi: 10.1186/s12967-019-2069-y (PMC6757394; doi:10.1186/s12967-019-2069-y)
Supplement: Supplementary file 2 — Additional file 2: Table S2. Clinicopathologic characteristics of all MBC patients (n = 2267). [file 12967_2019_2069_MOESM2_ESM.docx]

| **Table S2.** Clinicopathologic characteristics of all MBC patients (n=2267) | |
| --- | --- |
| **Patient characteristics** | **N(%)** |
| All Patients | 2267 |
| *Age at diagnosis* |  |
| <60 | 1096(48.3%) |
| ≥60 | 1171(51.7%) |
| *Ethnicity* |  |
| White | 1749(77.2%) |
| Black | 351(15.5%) |
| Others | 159(7.0%) |
| Unknown | 8(0.4%) |
| *Marital status* |  |
| Single | 325(14.3%) |
| Married | 1864(82.2%) |
| Unknown | 78(3.4%) |
| *Grade* |  |
| G1 | 106(4.7%) |
| G2 | 275(12.1%) |
| G3 | 1541(68.0%) |
| G4 | 99(4.4%) |
| Unknown | 246(10.9%) |
| *Estrogen receptor status* |  |
| Negative | 1747(77.1%) |
| Positive | 384(16.9%) |
| Unknown | 136(6.0%) |
| *Progesterone receptor status* |  |
| Negative | 1848(81.5%) |
| Positive | 277(12.2%) |
| Unknown | 142(6.3%) |
| *Stage TNM* |  |
| I | 530(23.4%) |
| II | 1384(61.0%) |
| III | 353(15.6%) |
| *Stage T* |  |
| T1 | 594(26.2%) |
| T2 | 1157(51.0%) |
| T3 | 368(16.2%) |
| T4 | 148(6.5%) |
| Unknown |  |
| *Stage N* |  |
| N0 | 1740(76.8%) |
| N1 | 368(16.2%) |
| N3 | 96(4.2%) |
| N4 | 63(2.8%) |
| *Axilla LN operation* |  |
| ANNS<=5 | 1161(51.2%) |
| ALND | 1106(48.8%) |
| *Breast operation* |  |
| Lumpectomy | 946(41.7%) |
| Mastectomy | 1321(58.3%) |
| *Chemotherapy* |  |
| Not done/unknown | 802(35.4%) |
| Done | 1465(64.6%) |
| *Radiotherapy* |  |
| Not done | 1181(52.1%) |
| Done | 1086(47.9%) |

SLNB: sentinel lymph node biopsy; ALND: axillary lymph node dissection
