# Supplementary figures and images for: The role of radiotherapy in metaplastic breast cancer: a propensity score-matched analysis of the SEER database
Source: J Transl Med. 2019 Sep 23;17:318. doi: 10.1186/s12967-019-2069-y (PMC6757394; doi:10.1186/s12967-019-2069-y)

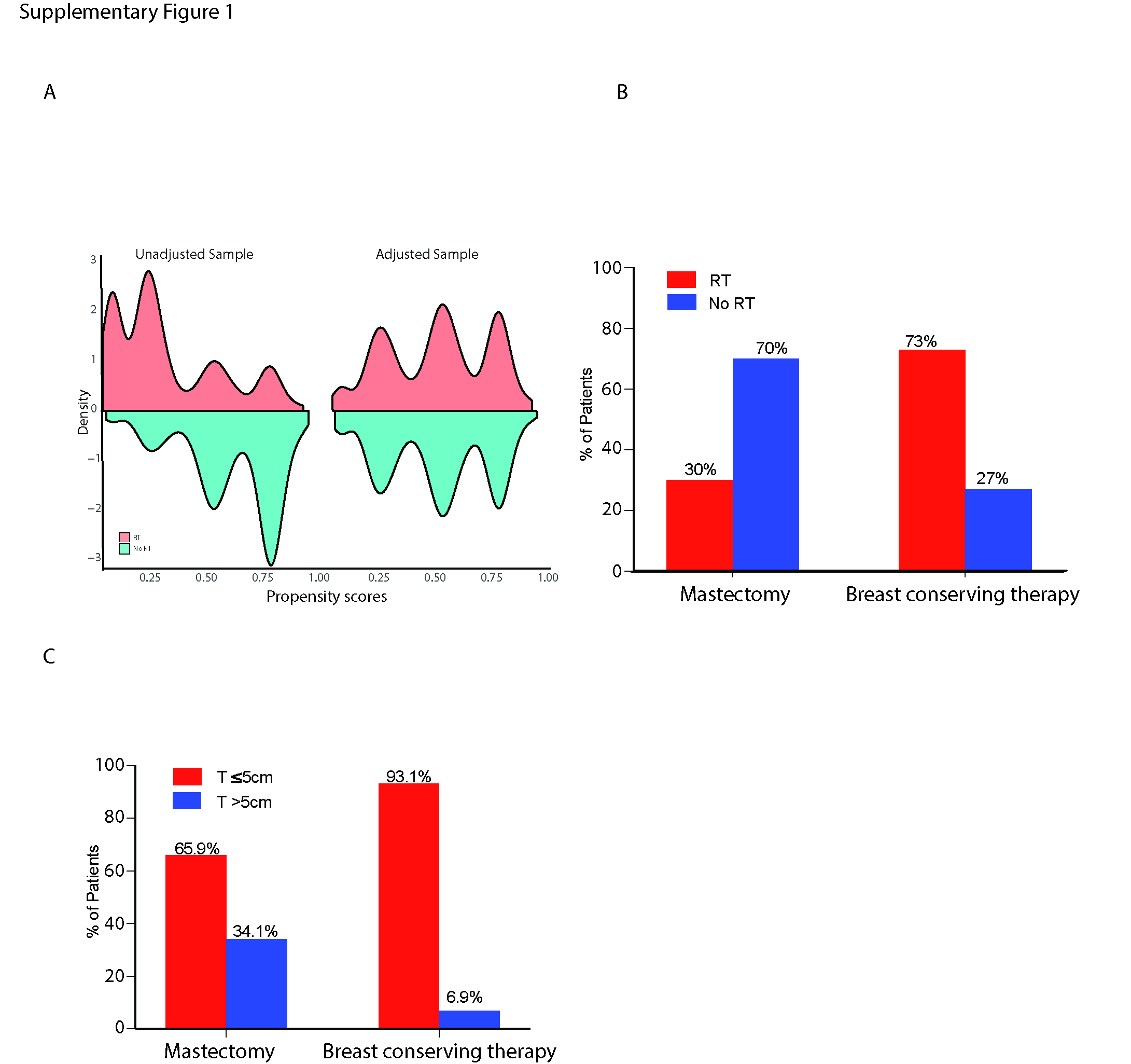

Supplement: Supplementary file 3 — Additional file 3: Figure S1. A. Plot of propensity score distribution before and after PSM; B. Breast operation by RT status before PSM; C. Breast operation by tumor size before PSM. [file 12967_2019_2069_MOESM3_ESM.tif]

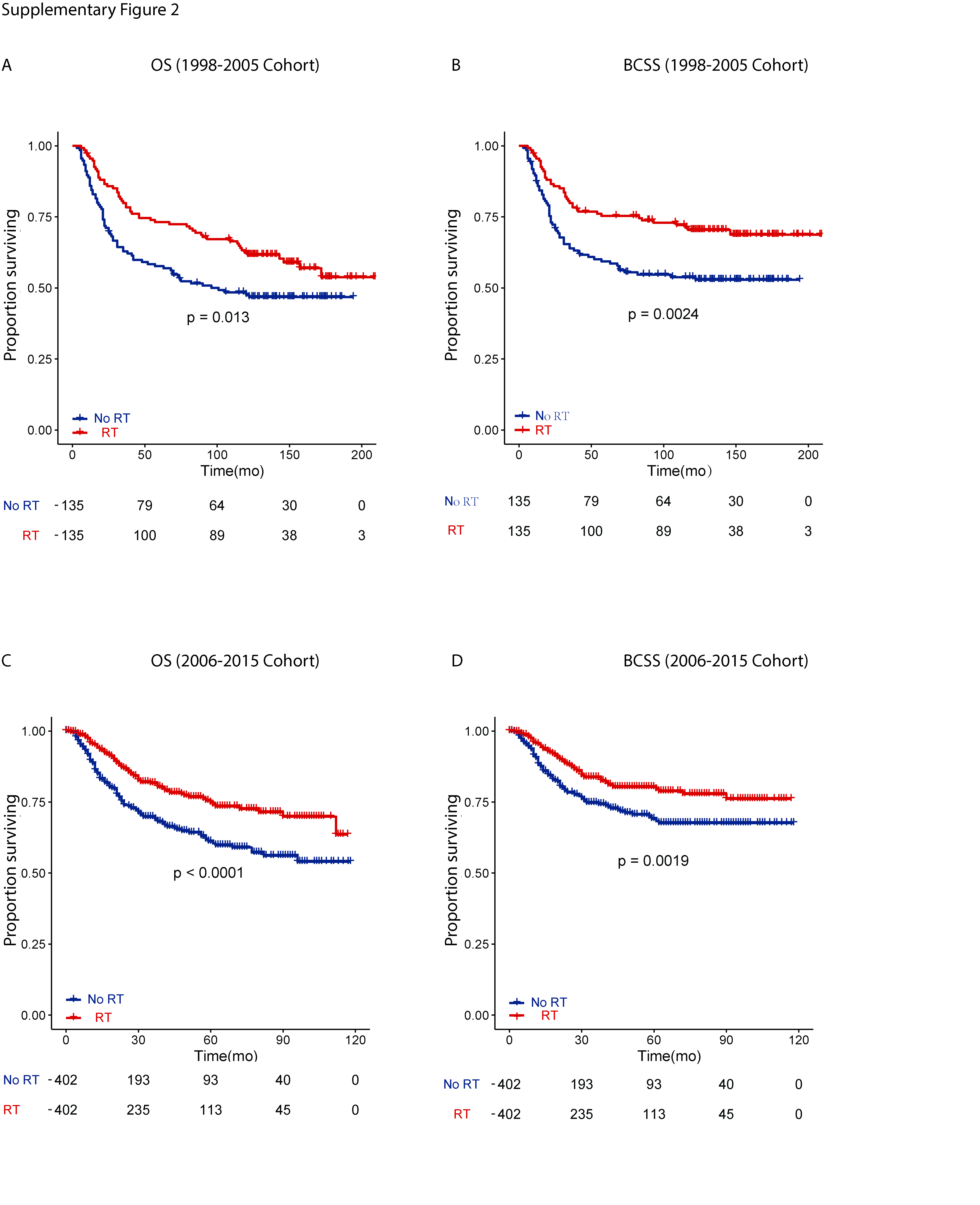

Supplement: Supplementary file 4 — Additional file 4: Figure S2. OS and BCSS of MBC patients displayed as Kaplan–Meier curve stratified according to RT in two subgroups. A. OS curves of RT cohort versus no RT cohort after PSM diagnosed from 1998 to 2005. B. BCSS curves of RT cohort versus no RT cohort after PSM diagnosed from 1998 to 2005. C. OS curves of RT cohort versus no RT cohort after PSM diagnosed from 2006 to 2015. D. BCSS curves of RT cohort versus No RT cohort after PSM diagnosed from 2006 to 2015. [file 12967_2019_2069_MOESM4_ESM.tif]

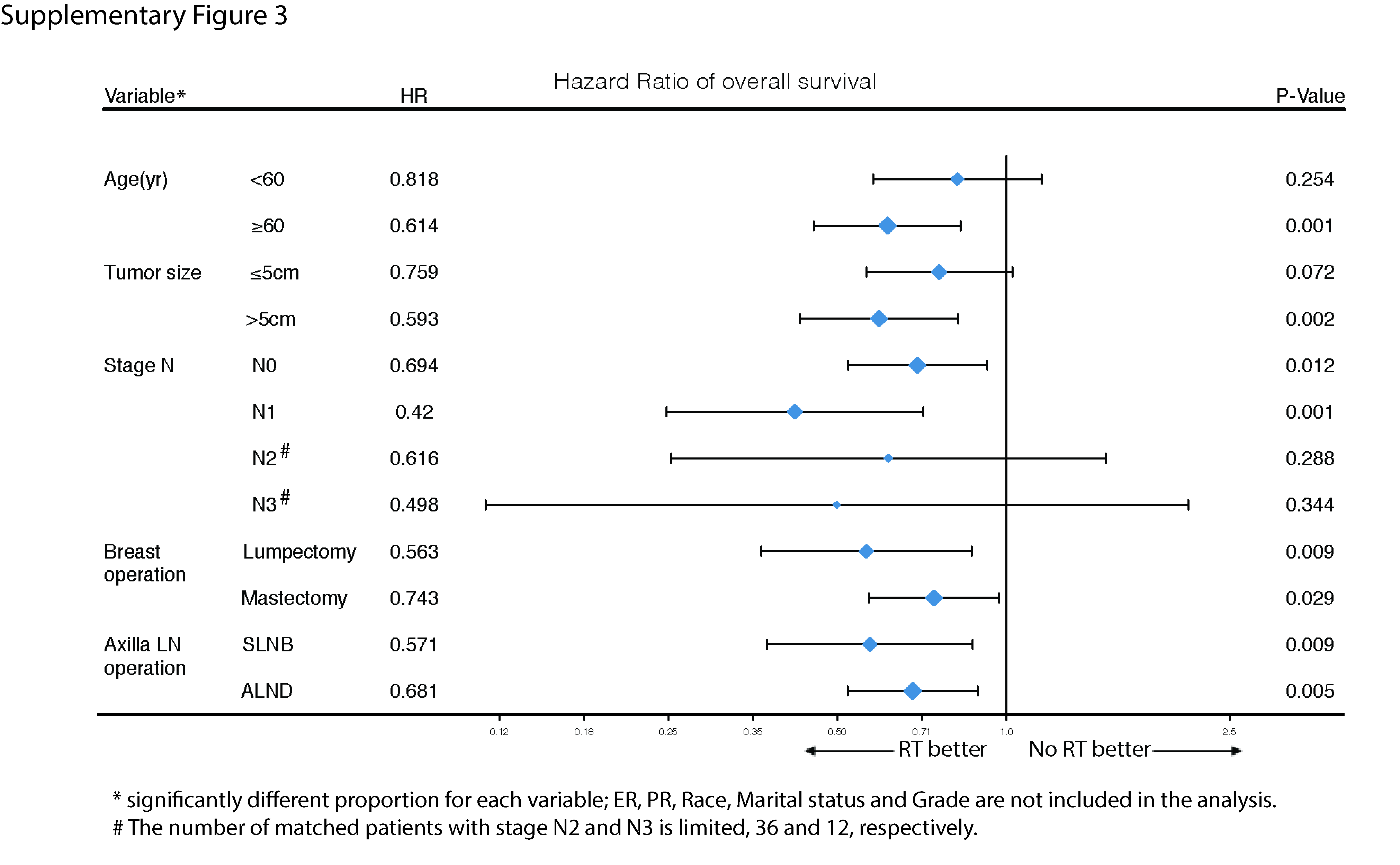

Supplement: Supplementary file 5 — Additional file 5: Figure S3. Hazard ratio and 95% confidence interval for OS according to receiving RT for different subgroups of patients for several variables: age, tumor size, N stage, type of breast operation, axillary LN dissection. [file 12967_2019_2069_MOESM5_ESM.tif]
